# Exploring Hormonal Pathways and Gene Networks in Crown Root Formation Under Stress Conditions: An Update

**DOI:** 10.3390/plants14040630

**Published:** 2025-02-19

**Authors:** Siarhei A. Dabravolski, Stanislav V. Isayenkov

**Affiliations:** 1 Department of Biotechnology Engineering, Braude Academic College of Engineering, Snunit 51, Karmiel 2161002, Israel; sergedobrowolski@gmail.com; 2 Institute of Agricultural and Nutritional Sciences, Martin Luther University Halle-Wittenberg, Betty-Heimann-Strasse 3, 06120 Halle, Germany; 3 Department of Plant Food Products and Biofortification, Institute of Food Biotechnology and Genomics, The National Academy of Sciences of Ukraine, Baidi-Vyshneveckogo Str. 2a, 04123 Kyiv, Ukraine

**Keywords:** crown roots, auxin, cytokinin, hormonal crosstalk, stress tolerance

## Abstract

Crown root (CR) initiation and development are crucial for the establishment of robust root systems in plants, contributing significantly to stress tolerance and overall growth. This manuscript explores the regulatory roles of key hormones and genes involved in CR formation, with a focus on their interactions under conditions of drought and salt stress. Cytokinins (CK) act as a negative regulator of CR development, while auxin (AUX) serves as a positive driver, facilitating cellular growth and division. *Wuschel-related homeobox (WOX)* genes, particularly *OsWOX11*, play a central role by integrating CK and AUX signalling to regulate downstream targets such as *OsCRL1* and auxin biosynthetic pathways. Other hormones, including jasmonic acid (JA) and gibberellin (GA), display context-dependent effects, modulating CR initiation based on environmental conditions. Critical genes like *OsESG1* and *OsFBX257* have been associated with improved drought resilience, interacting with proteins and kinases such as *OsGF14b*/c and OsCDPK1. Despite progress, significant challenges remain in mapping the full extent of hormonal crosstalk and gene regulation under stress conditions. This manuscript emphasises the need for future studies to incorporate comprehensive multi-omics approaches, expand the exploration of stress-related hormones like abscisic acid (ABA), and leverage advanced gene-editing techniques. Addressing these gaps will enhance our understanding of CR development and contribute to the development of crops with greater resistance to environmental stresses.

## 1. Introduction

Plants are confronted with a highly complex environment, and their root systems are crucial for overcoming these challenges. In addition to providing structural support to the plant’s above-ground parts, roots facilitate the uptake of water and essential nutrients required for growth. They also function as sensors, detecting soil properties such as moisture, nutrient content, and the presence of toxic elements. Root systems undergo continuous modification, forming and extending new roots throughout a plant’s lifecycle, thereby enabling optimal adaptation to varying environmental conditions, including both biotic and abiotic stressors [1,2]. By understanding the processes that regulate root patterning and identifying the genes responsible for post-embryonic root formation, breeders can enhance crops’ resilience to abiotic stresses [3,4,5].

Plant root systems can be categorised into two main types based on their developmental origin and branching characteristics: taproot systems and fibrous root systems. Taproot systems, typically found in dicotyledons such as *Arabidopsis thaliana* (L.) Heynh., *Solanum lycopersicum* L. (tomato), and *Pisum sativa* L. (pea), are characterised by a dominant primary root with lateral roots and root hairs. Fibrous root systems, on the other hand, are common in monocotyledons such as *Oryza sativa* L. (rice), *Triticum aestivum* L. (wheat), and *Zea mays* L. (maize) [6,7]. In taproot systems, the primary root plays a central role, with smaller lateral roots and root hairs developing from it. Fibrous root systems are more complex, consisting of a dense network of adventitious roots (also known as CR in cereals) that emerge from the stem, distinct from the primary and lateral roots [8,9]. In monocots, the embryonic root typically dies as the plant ages, leaving the adventitious roots to function as the primary root tissues [10].

Rice, as a model organism for monocots, provides an ideal platform for studying the molecular mechanisms governing both constitutive and adaptive root branching in fibrous root systems [3]. Unlike *A. thaliana*, which lacks adventitious roots, rice and other cereals develop numerous adventitious roots, making the genetic regulatory mechanisms identified in *A. thaliana* less relevant for understanding root development in monocots [11,12]. Plants exhibit remarkable plasticity in their growth throughout their lifetime, distinguishing them from mammals. In plants, two apical meristematic systems located at opposite ends of the plant body axis—the shoot apical meristem (SAM) and the root apical meristem (RAM)—are established during embryogenesis. These meristems are responsible for forming the entire plant body—both the root and aerial parts. The proper functioning of meristems depends on the presence of a small number of undifferentiated pluripotent stem cells, located in specialised environments called stem cell niches [13]. Adventitious roots (ARs) can develop directly from various aerial organs [14] and, depending on the status of the AR source cells, they may be directly fate-converted to AR founder cells by a root-inducing signal or may first need to acquire root competence through dedifferentiation [15].

In rice and other cereals, ARs constitute the main component of the root system, as the primary root originating from the embryonic RAM is short-lived. CRs are fundamental to fibrous root systems and are absent in taproot systems. In rice, CRs primordia develop from the innermost ground meristem cells, located adjacent to the peripheral cylinder of vascular bundles in the stem [16,17]. For simplicity and consistency, the term CRs will be used for these shoot-borne ARs in cereals, as they are part of the typical developmental programme of cereals [18]. However, it is important to note that ARs are also a common part of the natural root system of *A. thaliana* under normal growth conditions in soil [19]. Investigating the molecular regulation of CR development in rice is; therefore, essential for broadening our understanding of CR formation in other monocot species. Furthermore, identifying the key genes involved in rice root growth can support breeders in enhancing root structures for high-yield, nutrient-efficient, and stress-resilient crop varieties through tools such as genetic engineering and marker-assisted breeding [20].

CRs play critical roles in anchorage and soil resource acquisition during both vegetative growth and reproductive development [21]. All root types produce secondary roots (i.e., lateral roots), which originate from the pericycle of existing roots. Various studies have highlighted the transcriptomic, anatomical, and physiological complexity of the different root types in rice [22] and maize [23].

Among the numerous internal and external regulators, the plant hormones auxin and cytokinin are the most extensively studied [3,24,25]. Downstream of Auxin response factor1 (ARF1), CR formation is positively regulated by Crown rootless1/Adventitious rootless1 (CRL1/ARL1) and WOX11 [26,27]. In vascular plants, *Wuschel related homeobox* (*WOX*) transcription factor gene family is linked with stem cell regulation [28,29]. Among three identified superclades (the ancient clade, the intermediate clade, and the modern clade) [30], *WOX* genes from the intermediate clade (*IC-WOX* genes) have shown evolutionarily conserved functions in root organogenesis [31]. Rice WOX11 facilitates CR initiation by directly repressing the expression of *Response regulator2* (*OsRR2*), a type-A negative regulator of cytokinin signalling, in cooperation with the *Apetala2*/*Ethylene response factor* (*OsAP2*/*ERF*) transcription factor [18,32]. OsCRL5, another AP2/ERF transcription factor, up-regulates the expression of *OsRR1* and *OsRR2* independently of OsCRL1 [33]. Thus, CR initiation in rice relies on direct crosstalk between auxin and cytokinin signalling, mediated by coordinated transcription factor regulation.

In addition to hormonal regulation, various external factors, such as the availability of nutrients, can regulate CR development. Thus, soil Zn deficiency was linked to impaired CR initiation [34], while high ammonium supply increases CR formation but inhibits CR elongation [35]. In contrast, rice with well-developed CRs growing on soils contaminated with Cd accumulated more Cd, thereby affecting human health as Cd-contaminated rice. Accordingly, shoot Cd concentrations were positively correlated with root weight and CR number, and negatively correlated with root depth, suggesting that a lower CR number and a deeper root system would be more desirable traits for breeding low Cd-accumulating rice varieties [36].

With the advancement of functional genomics, there has been significant progress in deciphering the genetic control of root development in monocots through techniques such as mutant analysis, QTL mapping, and genome-wide association studies (GWASs). In this review, we briefly introduce the current understanding of CR development with a particular emphasis on the roles of key hormones such as CK, AUX, JA, and GA. Further, we focus on the leading role of modern omics techniques, primarily GWAS and transcriptomics, used for the identification of novel genes involved in CR development in rice/maize collections that are from various harsh environments and posses unique features, particularly high resistance to salt and drought stresses. Also, we delve into the genetic and molecular frameworks that mediate crosstalk between different plant hormones to regulate CR formation, highlighting critical genes like *OsWOX11*, *OsCRL1*, and stress-related genes such as *OsESG1* and *OsFBX257*. Finally, we identify existing research limitations and propose future directions that leverage advanced methodologies to enhance crop resilience.

## 2. Advances in Omics Approaches for Decoding Crown Root Development

Over the past two decades, ‘omics’ and modern biotechnological approaches have enabled the decoding of complex plant genomes, the assignment of functions to numerous previously unknown genes, and the development of genome-wide DNA markers. GWASs can identify genomic regions associated with phenotypic traits of interest using diversity panels sequenced and genotyped for single nucleotide polymorphisms (SNPs) or insertion/deletion variants (indels). This method is less labour-intensive and time-consuming compared to generating biparental populations for QTL mapping [37]. Various transcriptomics and metabolomics techniques (e.g., single-cell, spatial, and bulk approaches) have been extensively applied to study plant transcriptomes and metabolomes. These methods facilitate the systematic investigation of gene expression and metabolism within specific tissues and cell types at defined developmental stages, making them indispensable for studying plant quality and responses to environmental stimuli [38,39].

### 2.1. Genomic Insights and QTL Mapping in Crown Root Formation and Stress Resilience

A GWAS focusing on different root parameters in a collection of 180 rice accessions from Vietnam identified 88 loci with significant *p*- and *q*-values, specifically related to traits such as the number of CRs and the CRs per tiller. Of these 88 loci, 33 were located within genes with predicted functions. Some of these genes (or their homologues in *A. thaliana*) are involved in auxin regulation or root initiation and development, such as *AUXIN-INDUCED PROTEIN 4* (*OsIAA4*), an orthologue of *A. thaliana PHYTOCHROME-ASSOCIATED SERINE*/*THREONINE PROTEIN PHOSPHATASE1* (*FYPP1*), which regulates PIN1 protein activity, and an orthologue of *A. thaliana SORTING NEXIN2* (*SNX2a*), which regulates the endocellular transport of PIN2. Other identified genes included an orthologue of *A. thaliana POLYCYSTIN-1*, *LIPOXYGENASE*, *ALPHA-TOXIN AND TRIACYLGLYCEROL LIPASE 1* (*PLAT1*), which regulates lateral root development; an orthologous gene of *A. thaliana CYCLIN D6;1*, involved in lateral root initiation; and *OsMADS15*, which plays a role in CR development. The findings from this GWAS confirmed the universal role of known key regulators of CR formation and development while also identifying promising new candidate genes [40].

Another GWAS involving over 200 rice accessions from Southeast Asia characterised root morphological and anatomical traits related to productivity under drought stress and identified 59 candidate genes associated with root development. Notably, *Roothairless1* was linked with CR number, while *Histone acetyltransferase HAC703*, *Vacuolar cation/proton exchanger 2*, *Phosphate Starvation Response 3*, *Squamosa promoter-binding-like protein 3*, *plant U-box-containing protein 5*, and *Non-specific phospholipase C3* were associated with CRs per tiller [41]. A recent GWAS of 135 Japanese rice accessions mapped a quantitative trait locus for CR number on chromosome 4, identifying three candidate genes involved in CR number: a *Cullin* (*OsCUL3b*), a *Gibberellin 20 oxidase 8* (*OsGA20ox8*), and a *Cyclic nucleotide-gated ion channel* (*OsCNGC8*). These genes present opportunities for selection to enhance CR numbers in Japanese rice [42].

The implementation of bulk-segregant analysis has identified several rice genes involved in CR development under JA stimulation in Vietnamese rice. Further application of QTL sequencing pinpointed two rice receptor-like kinases (RLKs), *PUB54* and *PUB58*, within a genomic region on chromosome 10. Additionally, *Eukaryotic translation initiation factor 3 subunit L* (*eIF3l*) and *Mitogen-activated protein kinase kinase kinase 37* (*MAPKKK* 37) were identified as SNPs with high score indices, suggesting their roles in root development regulation under stress conditions [43].

Similarly, a GWAS conducted on 316 diverse maize inbred lines identified 113 genes related to CR angle, CR diameter, and CR number. Based on expression profiles, *GRMZM2G141205* (encoding an *IAA* family TF), GRMZM2G138511 (encoding *HSP* chaperone), and *GRMZM2G175910* (encoding *Cytokinin-O-glucosyltransferase*) were selected as potential candidate genes for CR development [44]. A GWAS of 339 global maize accessions identified three candidate genes associated with CR number under low phosphorus conditions: *Zm00001d031561* (encoding *bHLH62* TF), *Zm00001d001803* (encoding *Choline transporter-like protein*), and *Zm00001d001804* (encoding *MLO-like protein*). These genes, which contain binding sites for multiple transcription factors, including auxin response factors (ARFs) such as ARF4, ARF7, ARF10, and ARF16, are likely regulated by auxin [45].

Further research involving a GWAS and co-expression network analysis of 356 maize inbred lines from a global collection identified 26 high-priority candidate genes involved in CR growth and development. The exploration of these genes, including *GRMZM2G377215* (encoding a putative *GPI-anchored COBRA-like protein*), *GRMZM2G468657* encoding *Aspartic proteinase nepenthesin-1*), *GRMZM5G882427* encoding *Transferase family protein*), *GRMZM2G142779* (encoding *Actin cytoskeleton regulator during cell division)*, and *GRMZM2G151223* (encoding *Histidine Kinase 1*), could inform novel strategies to enhance drought tolerance, nutrient efficiency, and plant productivity [46].

### 2.2. Unravelling Crown Root Development Through Transcriptomic Analyses

The combined use of GWASs and transcriptome-wide association studies (TWASs) has significantly advanced the identification of genes responsible for root phenotypes. In a study involving 57 Japanese rice accessions, the integration of a GWAS and TWAS facilitated the identification of key genes linked to CR traits. GWAS analysis pinpointed *Equilibrative nucleoside transporter 1* (*OsENT1*) as a prime candidate gene, exhibiting a strong negative correlation with various root phenotypes, including CR length. Moreover, the TWAS highlighted *α-expansin 31* (*OsEXPA31EXPA31*) and *Squamosa promoter-binding-like protein 14* (*OsSPL14*) as candidate genes influencing CR diameter, while *Deceleraotor of internode elongation 1* (*OsDEC1*) emerged as a candidate for CR length regulation [47].

The regulatory network of rice CR formation, controlled downstream of *OsCRL1*, was characterised through a time-series transcriptomic analysis conducted after the induction of CR formation by CRL1 expression in a *crl1* mutant. This study identified 1753 differentially expressed genes (DEGs) that could be segmented into three distinct phases post-induction (from 3 to 21, 24 to 33, and 36 to 45 h after induction). Phase 1 DEGs were involved in DNA transcription regulation, sugar metabolism, cell organisation, and auxin and ethylene metabolism. In phase 2, the number of DEGs and associated biological ontologies notably decreased. Phase 3 showed an increase in DEGs, particularly those linked to protein degradation and cell wall organisation. Key genes such as *OsCRL6*, *OsIAA6*, *OsWOX11*, *OsQHB*, *OsIAA23*, and *Flattened shoot meristem* (*OsFSM*) were identified in this regulatory network, connecting CR initiation to root meristem maintenance and auxin signalling pathways [48].

Another transcriptomic analysis focusing on developing rice CR primordia identified 3975 DEGs, with approximately 30% expressed across three developmental stages. Stage-specific expression accounted for 10.5% at the early stage, 19.5% at the intermediate stage, and 12.8% at the late stage. Noteworthy genes implicated in these processes included transcription factors, chromatin remodelling factors, peptide growth factors, and cell wall remodelling enzymes. Down-regulated *Xyloglucan endotransglucosylase/hydrolases* (*OsXTH17*, *OsXTH1*, *OsXTH6*, and *OsXTH10*) were prominent, while *Plethora 3* (*OsPLT3*), *Phytosulfokine 1 PRECURSOR (OsPSK1), AUXIN RESPONSE FACTORS* (*OsARF5* and *OsARF24*), *Ethylene response factors* (*OsERF99* and *OsERF120*), several *OsExpansins*, and *OsIAAs* were among the most up-regulated genes in the early stage, with their expression diminishing at later stages [49].

A comparative transcriptomic analysis of embryonic primary roots, seminal roots, and post-embryonic CRs in the maize inbred line B73 identified 309 genes uniquely expressed in CRs, differentiating them from the other root types. Genes involved in signalling pathways, cell wall biosynthesis, cell organisation, secondary metabolism, and transport processes were notably enriched in primary and CRs. Crown and seminal roots shared an over-representation of cell wall biosynthesis and RNA metabolism genes. Furthermore, transcription factors of the *ARF* and *Homeodomain-leucine zipper* (HD-ZIP) families were overrepresented in primary and CRs, underscoring the broad role of auxin in root-specific expression profiles during maize root development [50].

### 2.3. Auxin-Driven Proteomic Modulations in Crown Root Formation

Recent proteomic analyses have reinforced the importance of auxin in translational and post-translational regulation during CR development. Through mass spectrometry, 334 proteins and 12 amino acids were identified in developing CR primordia and in mature CRs treated with auxin. These findings align with transcriptomic studies, highlighting the regulation of proteins involved in chromatin conformation, gene expression, and the cell cycle. Auxin-responsive regulation followed stage-specific patterns, affecting phosphorylation at 66 distinct phosphosites, which included key proteins such as Cyclin-dependent kinase G-2 (*OsCDKG;2*) and cell wall proteins. This suggests that auxin-dependent phosphorylation is essential for cell cycle progression and cell wall synthesis, facilitating root organogenesis [51].

Advancements in omics technologies, particularly transcriptomics and proteomics, have significantly improved our understanding of the molecular mechanisms underlying CR development. These approaches have allowed for the identification of key regulatory genes, proteins, and metabolites involved in CR initiation and response to environmental cues. Integrating multi-omics data has enabled researchers to map complex signalling pathways and interactions that control root architecture. The collective insights gained from omics studies are crucial for developing targeted approaches to enhance root traits, particularly for improving crop resilience under stress conditions. However, more comprehensive datasets and integrative analyses are needed to bridge the gaps in our knowledge and refine our understanding of the specific roles of various molecular components in CR development.

## 3. Regulatory Networks of Hormonal Control in Root Development

### 3.1. Hormonal Interplay: Cytokinin and Auxin Dynamics in Root Architecture

The antagonistic activities of auxin and cytokinin ensure proper root development through a balanced interplay that is crucial for root architecture formation. Exogenous auxin treatment using a TCSn-based cytokinin-responsive reporter [52] demonstrated cytokinin’s role in tissue differentiation during the development of rice CR primordia. Genetic experiments showed that endogenous auxin depletion stunted rice growth and led to the loss of apical dominance, while exogenous auxin supplementation rescued the CR phenotype, underscoring the significance of tissue-specific auxin–cytokinin interaction in root architecture formation [53].

**Figure 1:**
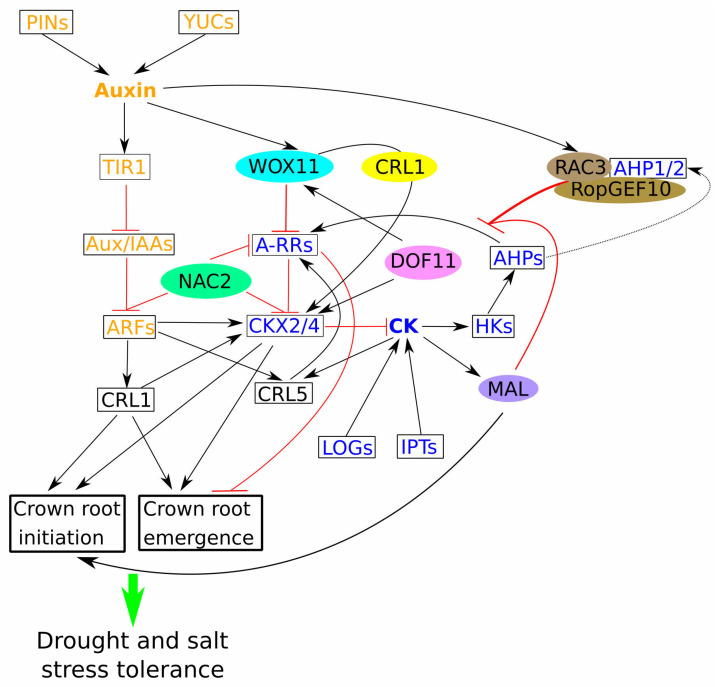
Auxin–cytokinin interplay in the regulation of crown root development in rice. Auxin (depicted in orange) is considered as a positive regulator, while cytokinin (depicted in blue) as a negative regulator of crown root development. NAC2 negatively regulates both auxin and cytokinin signalling pathways. DOF11 positively regulates WOX11 and CKX4. Additionally, WOX11 interacts with CRL1 to enhance CKX4 activation. Auxin-regulated RAC3 engages AHP1/2 in its complex with RopGEF10, thereby negatively regulating cytokinin signalling. Additionally, MAL acted as a negative regulator of cytokinin signalling and a positive regulator of crown roots initiation. Crown root initiation and crown root emergence have been associated with increased tolerance to salt and drought stress. Black arrows represent positive regulation; blunt red lines represent negative regulation. The green arrow represents the indirect effect.

Recent studies identified the OsRopGEF10–OsRAC3–OsAHP1/2 signalling module as another link between auxin and cytokinin during CR development [54]. Additionally, the OsNAC2 (NAM, ATAF, and CUC) transcription factor emerged as a signalling hub integrating auxin and cytokinin pathways (Figure 1). *OsNAC2* expression was observed in primary root tips, CRs, and lateral root primordia, indicating its involvement in root development. Knockdown of *OsNAC2* led to increased primary root length and CR numbers, whereas overexpression up-regulated CK biosynthesis genes (*OsIPT3*, *OsIPT5*, and *OsLOGL3*) and down-regulated CK degradation genes (*OsCKX4* and *OsCKX5*), resulting in elevated cytokinin levels. In *OsNAC2*-silenced lines, IAA biosynthesis genes (*OsYUCCA5* and *OsYUCCA6*) were up-regulated while IAA inactivation genes (*OsGH3.1*, *OsGH3.6*, and *OsGH3.8*) were down-regulated, indicating *OsNAC2*’s role in modulating IAA contents by directly influencing related genes [55]. Furthermore, OsNAC2 binds directly to the promoters of *OsGH3.6*, *OsGH3.8*, *OsARF25*, and *OsCKX4*. Analysis showed that *OsCRLs* and *OsCDKs* gene levels increased in *OsNAC2* knockdown and knockout lines but were reduced in overexpressing lines, confirming OsNAC2 as an integrator of auxin and cytokinin pathways, influencing cell division and root development.

The NAC family member OsNAC121 also modulates CR formation by controlling auxin transport and the auxin-to-cytokinin ratio. *OsNAC121* loss-of-function lines showed decreased CR numbers and primary root length, with auxin treatments failing to compensate for these defects. OsNAC121 interacts with TPL proteins (B8AKA4 and A2YRH5), forming a complex with *OsIAA10*, a core component of the auxin signalling pathway [56].

The intricate balance between CK and AUX is essential for the regulation of CR initiation and development. CK generally acts as a negative regulator, inhibiting root initiation, while AUX is a positive regulator that promotes root formation. The interaction between these hormones determines the spatial and temporal patterns of CR growth. Studies have highlighted the central role of CK signalling in modulating the expression of auxin transporters and responsive genes, which in turn affects auxin distribution and CR development. The balance between CK and AUX signalling is critical for maintaining optimal root architecture. Understanding how these hormones coordinate root growth can help guide strategies to modify root development for improved stress resilience and nutrient uptake.

#### The WOX Family: Integrators of Hormonal Signalling in Root Development

OsWOX11 is a critical regulator of CR growth, lateral root initiation, root hair formation, and responses to abiotic stress in rice. Interestingly, the expression level of *HmWOX2* was 7 times higher under osmotic stress and *HmWOX10* was 3.5 times higher under salinity in the roots of halophytic barley *Hordeum marinum*, suggesting a crucial role of this type of protein in the regulation of environmental stress adaptation [57]. Transcriptomic analysis of *wox11* root tips under cytokinin treatment and drought stress identified 664 DEGs related to rice root development, cytokinin homeostasis, stress responses, and redox metabolism [58]. Additionally, OsCRL1’s interaction with OsWOX11 enhances *OsCKX4* activation during rice root emergence and elongation, as evidenced by severe root phenotypes in *wox11*/*crl1* double mutants and altered cytokinin levels in *OsCKX4* knockout plants (Figure 1). *OsCKX4* overexpression partially rescued CR phenotypes in *crl1* and *wox11* mutants, further supporting cytokinin’s negative regulatory role and OsWOX11’s involvement in CR development [59].

Auxin’s critical role in rice root development is also highlighted by the overexpression of the *OsYUC1* gene that encodes a key enzyme in auxin biosynthesis, leading to CR proliferation in a *OsWOX11*-dependent manner. Conversely, *OsYUC1* disruption repressed *OsWOX11* expression and diminished CR development [60].

Ethylene has also been implicated in CK/AUX crosstalk, influencing rice CR development. OsWOX11 interacts with OsERF3 to repress type-A *OsRR2*, impacting cytokinin signalling and modulating CR development [32]. The *wox11* mutant showed reduced root hair length and increased drought sensitivity, whereas *OsWOX11* overexpression led to longer root hairs and improved drought resistance [61]. Soil compaction-induced ethylene accumulation promotes CR primordia initiation via the Ethylene insensitive 3-like 1 (OsEIL1) protein, which activates *OsWOX11* expression. Correspondingly, *wox11* mutants displayed an impaired ethylene response and reduced CR initiation under soil compaction [62].

The *WOX* gene family, particularly *OsWOX11*, serves as a central hub in rice CR development by integrating CK and AUX signals. *Os*WOX11 positively regulates CR initiation and development by promoting the differentiation of founder cells into root primordia. This regulation involves AUX, which enhances *Os*WOX11 expression, while CK modulates this process by influencing auxin transport and response. The interaction between *OsWOX* genes and hormone signalling pathways underscores their critical role in root system architecture. By orchestrating the hormonal crosstalk, *OsWOX* genes ensure the proper balance required for CR formation. The importance of *Os*WOX11 in the developmental framework suggests its potential as a target for genetic modification to improve root architecture and stress adaptability in crops.

### 3.2. CK-Mediated Regulation of Root Growth and Stress Adaptation

Recent research has elucidated the critical function of CK in modulating key genes involved in rice development. The *Grain number 1A/Cytokinin oxidase 2* (*Gn1A/OsCKX2*) gene is notably down-regulated in rice varieties characterised by heavy panicles, contributing to increased waterlogging resistance. Knockout studies of *Gn1A*/*OsCKX2* have confirmed notable enhancements in culm diameter, expanded vascular bundles, and a greater breaking force in comparison to WT plants. The expression of *Gn1A*/*OsCKX2* in tissues adjacent to the CR, particularly the upper root sections and lateral roots where vascular development occurs, underscores its role in root development. The knockout mutant demonstrated an accumulation of cytokinin-free bases and ribosides in the CR tip, leading to accelerated adventitious root formation, indicative of active cytokinin metabolism during CR development (Figure 1). Furthermore, the type-A OsRRs (*OsRR1*, *OsRR4*, *OsRR6*, and *OsRR10*) were down-regulated in the *Gn1A*/*OsCKX2* knockout line, suggesting that loss of this gene promotes CR development by increasing active cytokinin levels without adversely affecting rice quality [63].

The *Meristem activityless* (*MAL*) gene, containing a RING-H2 finger domain (RFD), plays a pivotal role in ensuring cell viability within the meristem during root primordia initiation, driven by CK and reactive oxygen species (ROS) interactions. Knockdown *MAL* transgenic rice exhibited a reduced CR length and fewer CRs, alongside diminished cell division rates, correlating with *MAL’s* expression patterns (Figure 1). Transcriptomic data indicated that exogenous CK exposure in *MAL* knockdown lines altered the expression of 1179 genes, including those related to CK metabolism and signalling. Affected genes comprised *Cytokinin dehydrogenase precursor*, *Cis-zeatin O-glucosyltransferase*, *RR1-9*, *CKX1-7*, and *Isopentenyl transferase gene 5* (*IPT5*). Gene clusters tied to cell wall metabolism and redox regulation were also implicated, confirming MAL as a regulator of CK- and ROS-mediated meristem cell viability [64].

*DNA binding with one finger 11* (*OsDOF11*) has been identified as essential for CR development. Mutants deficient in *OsDOF11* displayed fewer CRs, impaired nitrogen metabolism, and reduced CK and auxin levels. This was accompanied by lowered expressions of *OsWOX11*, *OsRR2/3*, and *OsCKX4* (Figure 1). Application of exogenous CK to these mutants rescued CR formation, demonstrating *OsDOF11*’s role in facilitating CR initiation through CK signalling [65].

CK-regulated genes play a significant role in CR development by modulating key processes such as cell division, differentiation, and response to environmental signals. CK primarily acts as a negative regulator of CR initiation, and its influence is mediated through downstream targets like ARRs and other CK-responsive transcription factors. The modulation of CK-responsive genes affects auxin distribution and signalling, thereby influencing CR formation. The balance between CK and auxin-responsive genes is essential for determining root architecture. By understanding the network of CK-regulated genes, researchers can develop strategies to modulate root growth patterns to achieve desirable traits, such as enhanced stress tolerance or improved nutrient acquisition.

### 3.3. Auxin and Stress Resilience: Insights from Root Gene Networks

The Pin-formed (PIN) auxin efflux transporters are integral to the polar transport of auxin, a key factor in plant development [66]. Mutations in the *OsPIN1* gene family (e.g., pin1a *pin1b pin1c* and *pin1a pin1b pin1d*) resulted in notable phenotypic defects, including shorter, curled shoots, diminished crown and lateral root formation, and primary roots with elongated root hairs. The quadruple mutant (*pin1a pin1b pin1c pin1d*) was non-viable. Altered expression profiles of auxin signalling-related genes (*OsIAAs*, *OsARFs*, and *OsRRs*) and CR regulatory genes (*OsCRL1*/*OsARL1*, *OsCRL4*, *OsCAND1*, *OsERF3*, and *OsSPL3*) were evident in these mutants, reinforcing the functional redundancy of *OsPIN1* paralogues in CR development (Figure 1) [67].

Auxin and ROS interactions are also crucial for rice CR formation. Treatment with potassium iodide (KI) affected ROS homeostasis, altering root architecture by reducing the CR length and number. Gene expression analyses indicated that auxin-responsive ROS homeostasis genes, such as *Peroxidases* (*POX*), *Glutathione-S-transferases* (*GST*), *Glutathione reductases* (*GR*), and *Thioredoxins* (*TRX*), were impacted during CR primordia initiation. Auxin treatment partially reversed KI-induced effects, restoring CR traits and up-regulating expression in 9 *POX*, 12 *GST*, 1 *GR*, and 1 *TRX* genes, while down-regulating 12 *POX* and 1 *GST* genes, highlighting the complex auxin–ROS interplay in CR primordium development [68].

Auxin is a positive regulator of CR initiation and development, with AUX-regulated genes playing a crucial role in facilitating root growth and patterning. These genes are involved in auxin biosynthesis, transport, and signalling pathways that control CR primordium initiation and emergence. AUX-regulated genes, including PIN and ARF family members, coordinate cell division and differentiation within root tissues. The precise regulation of these genes ensures the formation of a robust root system capable of adapting to various environmental conditions. By dissecting the roles of AUX-regulated genes, researchers can better understand how to manipulate auxin pathways to improve root development and stress adaptability in crops.

### 3.4. Gibberellins and Jasmonates: Hormonal Interplay in Crown Root Development

**Figure 2:**
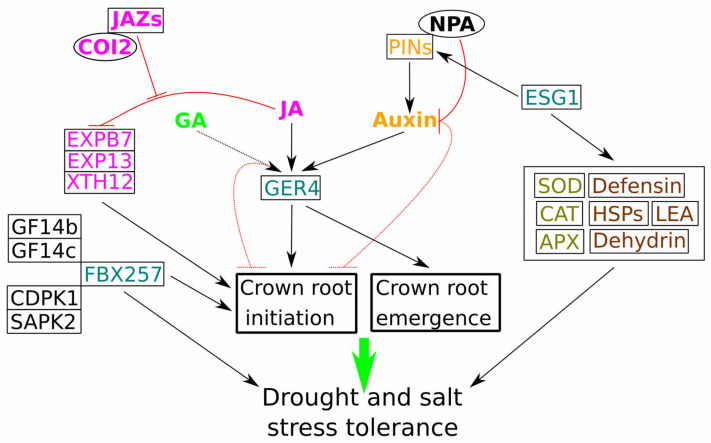
Crosstalk of various hormones and genes in the regulation of rice crown roots development. GER4 is a central gene integrating GA, JA, and auxin signalling pathways. JA negatively regulate crown root development through negative regulation of the cell elongation genes *OsEXPB7*, *OsEXP13*, and *OsXTH12*, with JA repressors *JAZs* and *COI2* acting in the opposite way. NPA treatment negatively regulated crown root emergence by affecting auxin polar transport through its effect on PINs. Similarly, *ESG1* altered the expression of auxin metabolism-related genes and transporters, negatively affecting crown roots development. Also, ESG1 regulated a wide range of antioxidant enzymes and stress-related proteins, which positively correlated with abiotic stress tolerance. FBX257 interacted with 14-3-3 proteins (GF14b and GF14c) and kinases (CDPK1 and SAPK2) to increase tolerance to drought. Black arrows represent positive regulation; blunt red lines represent negative regulation. The green arrow represents the indirect effect.

Recent research has identified that OsGER4, a rice Germin-like protein, promotes CR development in a JA-dependent manner. Notably, *OsGER4* knockout in Kitaake rice plants resulted in fewer CR and fewer CR primordia when subjected to long-term JA treatment. Under JA treatment, *OsGER4* was expressed throughout the root system, with high concentrations in actively growing regions of CR and LR. However, treatment with NPA, an inhibitor of polar auxin transport, altered *OsGER4* expression patterns, confining it to the pericycle, endodermis, and epidermis of CRs. This shift was associated with blocked LR production and reduced CR numbers (Figure 2) [69].

Further studies demonstrated that exogenous gibberellin application reduced the number of CRs in an OsGER4-dependent manner. Specifically, gibberellin treatment increased the number of CR in *osger4* mutant plants, while the overexpression of *OsGER4* had the opposite effect. *OsGER4* was strongly expressed in the exodermis, epidermis, sclerenchyma, endodermis layers of the CR, vascular bundles, and throughout LR primordia under GA_3_ treatment [70]. Additionally, recent findings linked *OsGER4* expression to the auxin signalling pathway in *oscrl1* mutant plants. *OsGER4* was expressed in the initiation and emergence zones of CRs and LRs under auxin treatment. Moreover, *osger4* plants produced fewer CRs than wild-type plants under auxin treatment, suggesting that OsGER4’s localisation to plasmodesmata may influence auxin flow, thus regulating CR development (Figure 2) [71]. Collectively, these findings position OsGER4 as a central protein integrating auxin, gibberellin, and JA signalling pathways in the regulation of CR initiation and development.

Conversely, another study reported that JA treatment inhibited CR growth in wild-type Kitaake rice plants. This effect implicated the *F-box Coronatine insensitive* (*COI1/2*) genes and *Jjasmonate zim domain* (*JAZ5/8*) transcriptional repressors in CR growth regulation. The inhibitory effect of JA on CR growth was less pronounced in *oscoi2* mutants, which showed expression in CR tips. In *oscoi2* backgrounds, the induction of JA biosynthesis (*OsAOC*) and signalling (*OsJAZ5*, *OsJAZ8*, and *OsMYC2*) genes by exogenous JA treatment was diminished compared to the wild-type plant, whereas this effect was not observed in *oscoi1* mutants. Furthermore, cell elongation-related genes (*OsEXPB7*, *OsEXP13*, and *OsXTH12*) were less repressed by JA treatment in *oscoi2* CRs compared to WT and *oscoi1* plants (Figure 2). These results suggest that *OsCOI2* is necessary for JA-dependent signalling in rice CRs [72].

The interplay between GA and JA provides additional layers of regulation for CR development. GA generally acts as a negative regulator of CR initiation, with exogenous application reducing CR numbers through mechanisms involving OsGER4. Conversely, JA can have both positive and negative effects; in some studies, it has promoted CR development, while in others, it has inhibited growth, depending on genetic backgrounds and specific signalling components. JA’s role is mediated by components such as OsCOI2 and JAZ proteins that influence the expression of cell elongation-related genes. The modulation of CR development by GA and JA highlights the complexity of hormonal regulation, where context-specific interactions determine the outcome. Understanding these dynamics can inform breeding programmes aimed at improving root traits for drought tolerance and nutrient efficiency.

### 3.5. OsESG1 and OsFBX257 in Root Development: Linking Auxin Transport, Architecture, and Stress Tolerance

OsESG1, an S-domain receptor-like kinase, plays a significant role in early rice CR development and drought response. Knockdown of *OsESG1* resulted in fewer CRs and a reduced shoot length compared to wild-type plants. The expression of auxin-related genes was altered in knockdown lines: *OsIAA1*, *OsIAA22*, and *OsIAA16* were up-regulated, and *OsPIN1b*, *OsPIN2*, and *OsPIN10a* were up-regulated, while *OsPIN9* was down-regulated. Consequently, polar auxin transport (PAT) was disrupted, impairing CR primordium initiation and formation. Under osmotic stress, mutant plants displayed reduced antioxidant enzyme (SOD, CAT, and APX) activity, leading to increased ROS and MDA accumulation. Additionally, the accumulation of stress-related proteins, such as heat shock proteins (HSPs), late embryogenesis abundant (LEA) proteins, dehydrin, and defensin, was absent in mutants under stress (Figure 2). These findings suggest that OsESG1 is involved in regulating CR initiation and development by modulating auxin response and distribution, and it contributes to stress tolerance by controlling antioxidant activity and stress-responsive gene expression [73].

*OsFBX257*, an F-box gene involved in the SCF-type E3 ubiquitin ligase complex, was shown to modulate root architecture and enhance drought stress tolerance in rice. This modulation optimises the plant’s root system by promoting deeper and more extensive root growth, allowing for better water absorption and retention under arid conditions. Enhanced root architecture contributes to improved stability and nutrient uptake, crucial for maintaining growth and productivity during periods of limited water availability. Knockdown of *OsFBX257* resulted in reduced total root length and depth, CR number, panicle size, and survival under drought stress, whereas overexpression led to opposite effects. Mechanistically, these outcomes may be due to OsFBX257’s interaction with the 14-3-3 proteins GF14b and GF14c, as well as its binding with kinases OsCDPK1 and OsSAPK2 (Figure 2). Therefore, OsFBX257 is a promising target for developing drought-tolerant rice varieties [74].

Genes such as *OsESG1* and *OsFBX257* contribute to the intricate regulatory network that governs CR development and stress response. OsESG1 plays a crucial role in CR initiation by modulating auxin transport and response, acting as a positive regulator of CR development. Additionally, it enhances stress tolerance by promoting antioxidant activity and the expression of stress-related proteins. On the other hand, *OsFBX257*, an F-box gene, positively influences root architecture by interacting with 14-3-3 proteins and kinases, which enhances drought stress tolerance. These findings underscore the importance of non-hormonal regulatory proteins in shaping root architecture and stress adaptability. The study of such genes provides valuable insights into breeding strategies for crops with improved root systems that support resilience under challenging environmental conditions.

## 4. Limitations and Directions for the Future Research

Current research on CR initiation and development has revealed critical insights into the regulatory pathways and genetic factors involved. However, significant gaps remain, which hinder a complete understanding of how these processes can be optimised to enhance stress tolerance, such as resistance to drought and salt stress. One major limitation is the incomplete mapping of the signalling crosstalk between different hormonal pathways, such as CK, AUX, JA, and GA. While AUX is well-recognised as a positive regulator of CR formation, CK acts as a negative regulator, and the negative or context-dependent effects of JA and GA highlight the complexity of their interactions. The intricate interplay between these hormones under stress conditions requires further exploration to delineate their precise roles in enhancing root architecture and resilience.

Another challenge lies in understanding how key regulatory genes, such as *OsESG1* and *OsFBX257*, modulate CR development under varying environmental conditions. For example, while *OsFBX257* has been identified as a potential target for improving drought tolerance, its interactions with proteins such as GF14b and GF14c, and kinases like OsCDPK1, require deeper investigation. Moreover, the molecular mechanisms underpinning the antioxidant response and stress-protective proteins regulated by *OsESG1* remain only partially characterised.

The *WOX* genes, particularly *OsWOX11*, have emerged as pivotal players in the regulation of CR development. Acting as integrators of CK and AUX signalling, *OsWOX11* mediates the balance between these hormonal pathways, modulating downstream targets such as *CRL1* and auxin biosynthetic genes to coordinate CR initiation and growth. Despite significant progress, more detailed investigation is needed to elucidate how *WOX* genes contribute to stress responses, particularly under drought and salt stress, by facilitating adaptive changes in root architecture. Future research should focus on how the regulatory functions of *WOX* genes can be harnessed to enhance CR formation under drought and salt stress, potentially by modulating their expression or identifying interacting partners that facilitate stress adaptation.

To address these current limitations, advancing and diversifying research methods for studying CR development is essential. Integrating multi-omics approaches, such as transcriptomics, proteomics, and metabolomics, will be crucial for constructing comprehensive networks of hormone signalling and gene expression under stress conditions. Advanced gene-editing techniques, including CRISPR/Cas9 and base-editing tools, can be employed to validate the functional roles of regulatory elements and precisely modify key genes for enhanced stress tolerance.

Moreover, expanding research on signalling pathways beyond the traditional focus can provide deeper insights. Future studies should prioritise the examination of pathways involving ABA, ethylene, and salicylic acid, which are known to play significant roles in plant stress responses. Investigating how these hormones interact with CK and AUX under drought and salt conditions can reveal new regulatory nodes critical for CR adaptation. Additionally, identifying and characterising novel genes, such as *NAC* and *MYB* family transcription factors, and other stress-associated gene families will enhance the understanding of CR development.

High-throughput phenotyping tools, such as automated imaging systems and root architectural modelling, should be leveraged to monitor CR growth under varying environmental conditions. These technologies will enable researchers to correlate specific genetic modifications with phenotypic outcomes, thus accelerating the identification of genetic markers linked to improved stress resilience.

To advance the field, collaborative efforts in the phenotyping and high-throughput screening of CR traits under diverse environmental conditions will enable the identification of robust genetic markers for breeding programmes. Further research should explore how epigenetic modifications, such as DNA methylation and histone modifications, influence CR development and stress adaptation. Understanding the regulatory roles of small RNAs, such as microRNAs, in modulating gene expression during CR formation under stress conditions is also an essential future direction.

Addressing these current limitations with innovative methodologies and targeted research will pave the way for developing crop varieties with improved root systems capable of sustaining growth and productivity in adverse environmental conditions.

## 5. Conclusions

The study of CR initiation and development continues to reveal the intricate network of hormonal regulation and gene expression that underpins this vital process. Central to CR formation are the regulatory roles of hormones such as CK, AUX, JA, and GA. While CK acts primarily as a negative regulator of CR initiation, AUX functions as a positive driver of CR development by promoting cellular division and elongation. The interplay between CK and AUX is moderated by key genetic components, including *OsWOX* genes, which integrate these signals to mediate the expression of downstream targets like *OsCRL1* and auxin biosynthetic genes. This integration is crucial for coordinating the precise growth and adaptation of CRs, particularly under stress conditions.

Emerging research has identified other regulatory pathways that modulate CR responses, with JA and GA exhibiting context-dependent roles that can suppress or promote CR development based on environmental factors. Genes like *OsESG1* and *OsFBX257* have been highlighted for their potential to enhance drought tolerance, demonstrating interactions with kinases and proteins such as OsCDPK1 and GF14b/c. Despite significant strides in understanding these pathways, knowledge gaps remain, particularly concerning the complete signalling crosstalk under stress conditions and how CR regulatory networks adapt to drought and salt stress.

Future research should expand the scope of study to include hormones such as ABA, ethylene, and salicylic acid, which play substantial roles in stress responses. Integrating multi-omics approaches and advanced gene-editing technologies will be essential for mapping the complete landscape of hormonal and genetic interactions. Through such efforts, a more comprehensive understanding of CR development and its potential for enhancing crop stress resilience can be achieved.

## Data Availability

Not applicable.
